# Comparative muscle irritation and pharmacokinetics of florfenicol-hydroxypropyl-β-cyclodextrin inclusion complex freeze-dried powder injection and florfenicol commercial injection in beagle dogs

**DOI:** 10.1038/s41598-019-53304-0

**Published:** 2019-11-13

**Authors:** Guoqing Fan, Li Zhang, Yun Shen, Gang Shu, Zhixiang Yuan, Juchun Lin, Wei Zhang, Guangneng Peng, Zhijun Zhong, Lizi Yin, Hualin Fu

**Affiliations:** 1 Innovative Engineering Research Center of Veterinary Pharmaceutics, College of Veterinary Medicine, Sichuan Agricultural University, Chengdu, Sichuan 611130 China; 2 Institute of Traditional Chinese Medicine Pharmacology and Toxicology, Sichuan Academy of Chinese Medicine Sciences, Chengdu, Sichuan 610041 China

**Keywords:** Drug delivery, Drug development

## Abstract

Florfenicol (FF) is a novel animal-specific amidohydrin broad-spectrum antibiotic. However, its aqueous solubility is extremely poor, far below the effective dose required for veterinary clinic. Thus, FF is often used in large doses, which significantly limits its preparation and application. To overcome these shortcomings, the FF-hydroxypropyl-β-cyclodextrin (FF-HP-β-CD) inclusion complexes were developed using the solution-stirring method. The physical properties of FF-HP-β-CD were characterized. A comparison was conducted between FF and FF-HP-β-CD freeze-dried powder injection of their muscle irritation and the pharmacokinetics. The drug loading and saturated solubility of FF-HP-β-CD at 37 °C were 11.78% ± 0.04% and 78.93 ± 0.42 mg/mL, respectively (35.4-fold compared with FF). Results of scanning electron microscopy, differential scanning calorimetry, X-ray diffraction, and Fourier transform infrared showed that FF was entrapped in the inner cavity of HP-β-CD, and the inclusion complex formed in an amorphous state. In comparison with FF commercial injection, FF-HP-β-CD increased the elimination half-life (*t*_*1/2β*_), transport rate constant (*K*_10_, *K*_12_, *K*_21_), and maximum concentration (*C*_*max*_) after intramuscular injection in beagle dogs. Conversely, it decreased the distribution half-life (*t*_*1/2α*_), absorption rate constant (*K*a), apparent volume of distribution (*V1/F*), and peak time (*T*_*max*_). These results suggest that FF-HP-β-CD freeze-dried powder injection is a promising formulation for clinical application.

## Introduction

Florfenicol (FF), also known as fluthiracnamline, is chemically named D(+)-threo-1-p-sulfonylphenyl-2-dichloroacetamido-3-fluoropropanol. FF is an excellent broad-spectrum antibiotic for animals with broad antibacterial spectrum, wide distribution in the body, and with safe and efficient features; particularly, it does not potentially cause aplastic anemia and without teratogenic, carcinogenic, and mutagenic effects^1^. FF is extensively used clinically for the treatment of bacterial diseases in livestock and poultry caused by susceptible strains. Chloramphenicol was banned from use as an animal-derived food for animals; thus, the market demand for FF has been further expanded. To date, FF has been used extensively as the main force for anti-infective drugs in various countries, and the amount of FF is tens of thousands of tons per year worldwide^2^. However, the bioavailability of FF is severely affected with an increased cost of the drug owing to the poor water solubility. Therefore, improving the water solubility of FF is of great importance for clinical application.

The chemical methods used to increase FF solubility include the synthesis of FF phosphate, FF sulfonate, and FF succinate^1^. However, the preparation conditions of FF phosphate are reported to be complicated and are unsuitable for industrial production. Although succinic prodrugs increase the water solubility of the drug, succinic prodrugs reduce its biological activity. In addition, other problems occur, such as low yield, poor stability, and numerous byproducts. The physical methods used for FF solubilization include the addition of cosolvents, micronization, nanoemulsion, *in-situ* gel, β-cyclodextrin (CD) inclusion complexes, and solid dispersion nanoparticles^3–6^. Although, reports are available on FF-β-CD and FF-hydroxypropyl (HP)-β-CD inclusion complexes, most of them were reported only for the preparation methods and their physical properties; the *in vivo* evaluation is rarely studied^7,8^. These methods can increase the solubility of FF to a certain extent; however, meeting the needs of clinical use as a commonly used preparation is difficult.

At present, the commonly used FF preparations in the clinic include powders, injections, solutions, and premixes. Moreover, with the large-scale and intensive farming of animal husbandry, oral administration, condiments, and drinking water have become the main administration modes. Therefore, premixes, powders, and oral solutions of FF are in considerable demand. However, for individual animals with special economic value, such as boars, breed bulls, and pets, people tend to devote other resources for diagnosis and treatment. Therefore, they are also the main consumers of FF injections. In addition to the advantages of the injection, such as less frequent administration, rapid onset, and suitability for animals with difficulty swallowing, injection also has a strong market demand. However, the commonly used FF injection (30%) is formulated with organic solvents (e.g., dimethylformamide, pyrrolidone, and propylene glycol) It can cause local muscle irritation and toxicity^5^. On the basis of the aforementioned reasons, developing water-soluble FF injection is of great importance.

HP-β-CD is a derivative of CD and thus has its advantages and has good water solubility at room temperature; moreover, it is stable to heat and nontoxic to kidneys. When administered parenterally, HP-β-CD is excreted in the urine and does not accumulate in the body. Moreover, it can promote the rapid release of the encapsulated substances. It also has low surface activity, low hemolytic activity, and safe for use. After the drug and HP-β-CD form an inclusion compound, it has the effect of increasing drug stability, preventing volatilization, reducing drug irritation, and increasing drug solubility. In this study, FF-HP-β-CD was prepared using the solution-stirring method to increase the solubility of FF. The characterization, muscle irritation, and pharmacokinetics of FF-HP-β-CD were investigated.

## Results and Discussion

### Preparation of FF-HP-β-CD

Commonly used inclusion complex preparation methods include grinding, ultrasonic, solution-stirring, freeze-drying, and spray-drying methods. The current study selected the solution-stirring method, and FF-HP-β-CD was successfully prepared on the basis of single-factor experiment and orthogonal design. On the basis of our research, the average drug loading is 11.78% ± 0.04%. Moreover, the prepared FF-HP-β-CD was observed to have a highly enhanced solubility of 78.93 ± 0.42 mg/mL at 37 °C, which is 35.4-fold compared with FF (2.23 ± 0.04 mg/mL) and is more than the reported solubility of 40.76 mg/mL^9^.

### Characterization of FF-HP-β-CD

#### Scanning electron microscopy (SEM)

**Figure 1:**
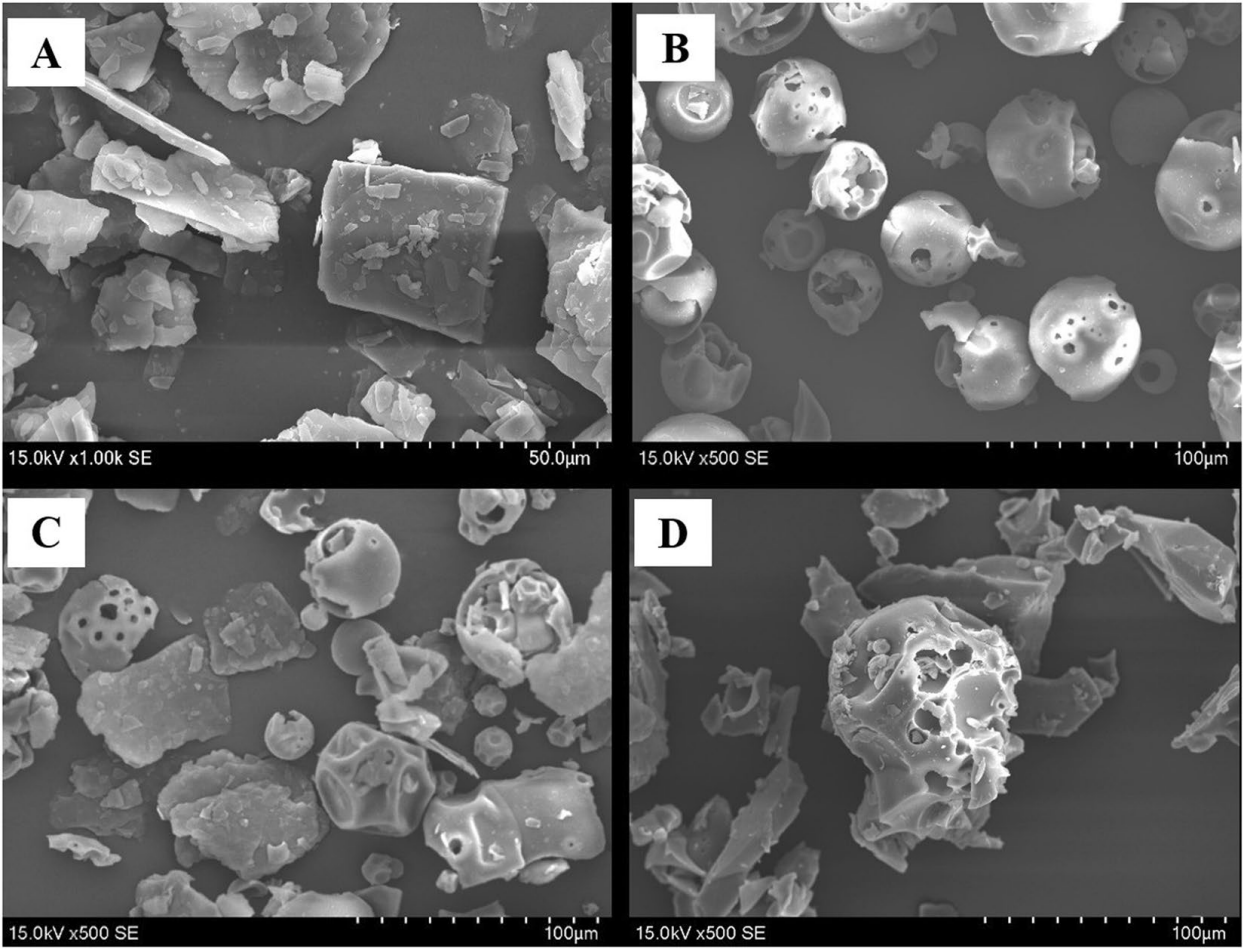
SEM images of FF (**A**), HP-β-CD (**B**), PM (**C**), and FF-HP-β-CD (**D**).

From the SEM images (Fig. 1), FF raw drug has an obvious flake crystal structure. HP-β-CD exhibits a typical spherical particle structure containing a cavity. The physical mixture (PM) of FF and HP-β-CD prepared in a molar ratio of 1:2 clearly shows flaky FF crystals and spherical HP-β-CD particles, indicating that the physical form of the two do not change. However, the morphology and crystal structure of FF-HP-β-CD lyophilized powder have undergone substantial changes. Neither a flaky crystal structure nor a distinct spherical structure has emerged but a new irregular amorphous state. Thus, the FF is embedded in the HP-β-CD cavity to form an amorphous-state inclusion complex.

#### X-ray diffraction (XRD)

**Figure 2:**
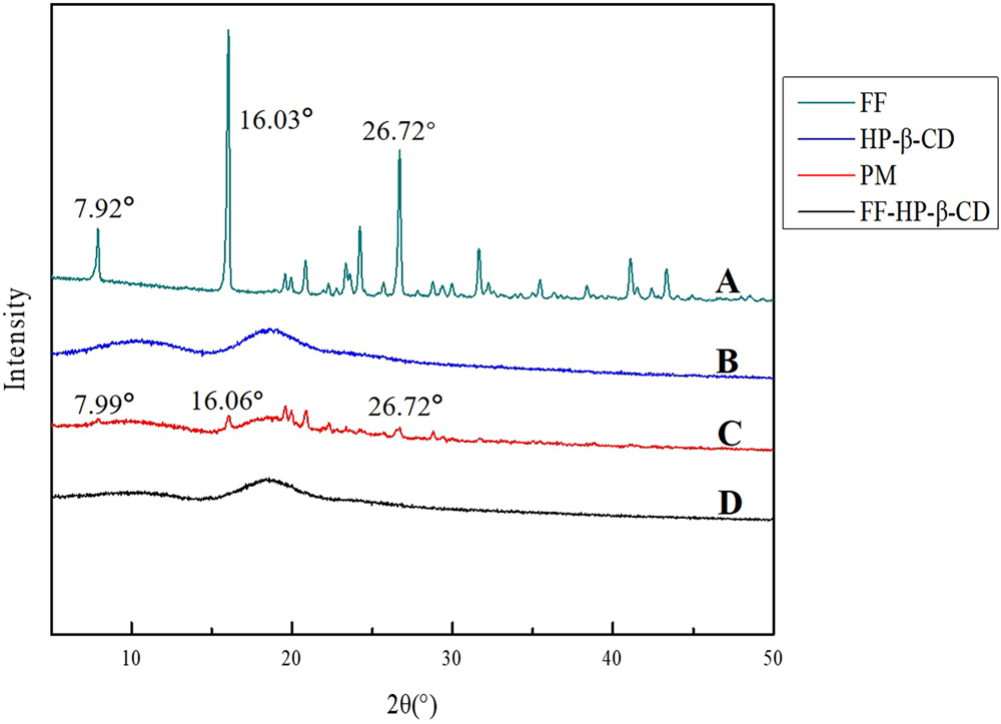
XRD patterns of FF (**A**), HP-β-CD (**B**), PM (**C**), and FF-HP-β-CD (**D**).

XRD analysis is a common method used to detect crystalline and amorphous forms. Figure 2 shows the XRD patterns of FF, HP-β-CD, PM, and FF-HP-β-CD, which are consistent with the literature^10^. FF has high-intensity characteristic diffraction peaks at different diffraction angles (2θ) at 7.92°, 16.03°, and 26.72°. Thus, the crystal form of FF-HP-β-CD shows no obvious characteristic diffraction peaks. It only exhibits two broad diffraction peak bands around 10° and 20°, indicating the amorphous state of HP-β-CD. PM shows a superposition of FF and HP-β-CD; however, the lower relative content of FF after mixing leads to a decrease in the intensity of the characteristic diffraction peaks. This finding indicates that the physical form of PM does not change. By contrast, the FF-HP-β-CD shows a similar result to the HP-β-CD diffraction peaks. The typical characteristic diffraction peak of FF completely disappears and belongs to the amorphous state, which may be related to the fact that FF is embedded in the HP-β-CD lumen. This result is consistent with the SEM results, and both reflect the amorphous state of the obtained FF-HP-β-CD.

#### Differential scanning calorimeter (DSC)

**Figure 3:**
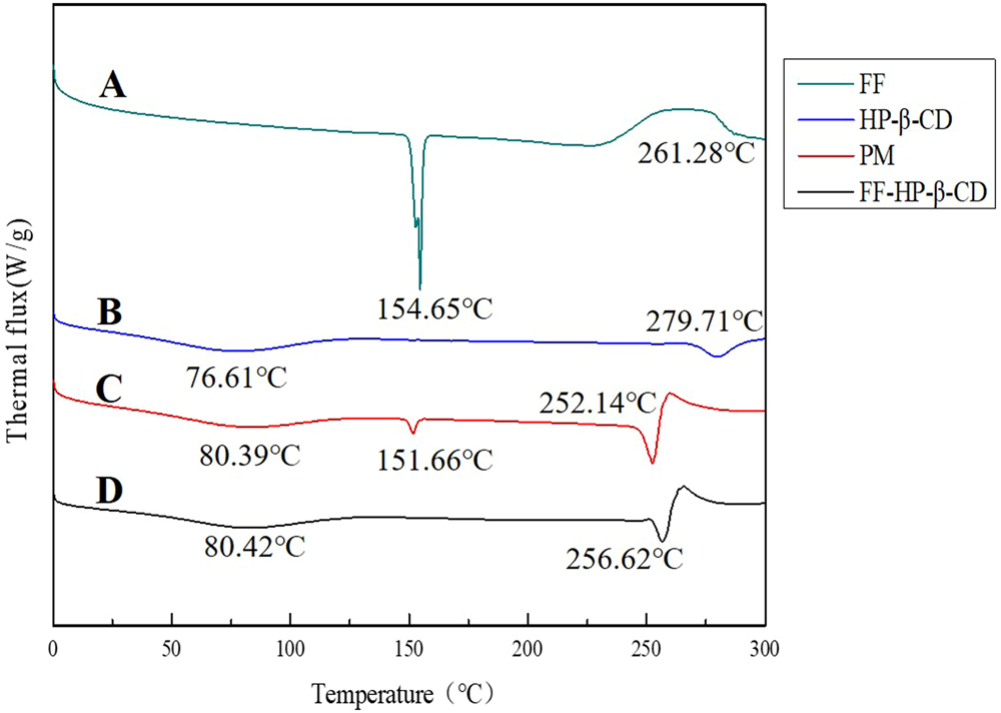
DSC thermograms of FF (**A**), HP-β-CD (**B**), PM (**C**), and FF-HP-β-CD (**D**).

In the DSC spectra (Fig. 3), FF has a characteristic endothermic peak (melting point peak) at 154.64 °C. This result is consistent with the results of Yang^11^. HP-β-CD has an endothermic peak at 279.71 °C. In addition, PM has two endothermic peaks, which serve as the superposition of those of FF and HP-β-CD. As expected, the spectra of FF-HP-β-CD are different from the PM. Only one characteristic endothermic peak of the HP-β-CD exists, with no characteristic endothermic peak of FF near 154 °C. This finding is most likely to occur because the melting point of the original drug FF is changed after it is embedded in the HP-β-CD cavity, thereby resulting in the disappearance of the original characteristic endothermic peak of the FF. Therefore, FF forms a clathrate with HP-β-CD and becomes a new amorphous phase.

#### Fourier transform infrared (FTIR)

**Figure 4:**
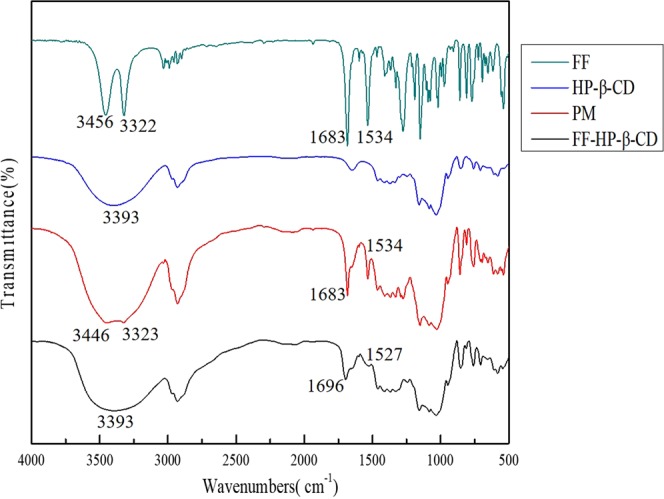
FTIR spectra of FF (**A**), HP-β-CD (**B**), PM (**C**), and FF-HP-β-CD (**D**).

Figure 4 illustrates the FTIR spectra of FF, HP-β-CD, PM, and FF-HP-β-CD. FF shows an –OH stretch band at 3456 cm^−1^, a –C=O band at 1683 cm^−1^, a –CN stretch band and –NH bending at 1534 cm^−1^, and a –NH stretch band and –CN bending at 1274 cm^−1^. This result is consistent with the literature^12^. HP-β-CD has an –OH stretch band at 3393 cm^−1^. The infrared spectrum of the PM is a superposition of the FF and HP-β-CD spectra. Different from PM, the four characteristic peaks of FF significantly weaken or disappear in the infrared spectrum of the FF-HP-β-CD. This finding indicates the formation of FF-HP-β-CD. The dichloromethyl (–CHCl2) structure side of FF can be further determined to be embedded in the HP-β-CD cavity, combined with the chemical structure of FF.

#### ^1^H nuclear magnetic resonance (^1^H-NMR)

**Figure 5:**
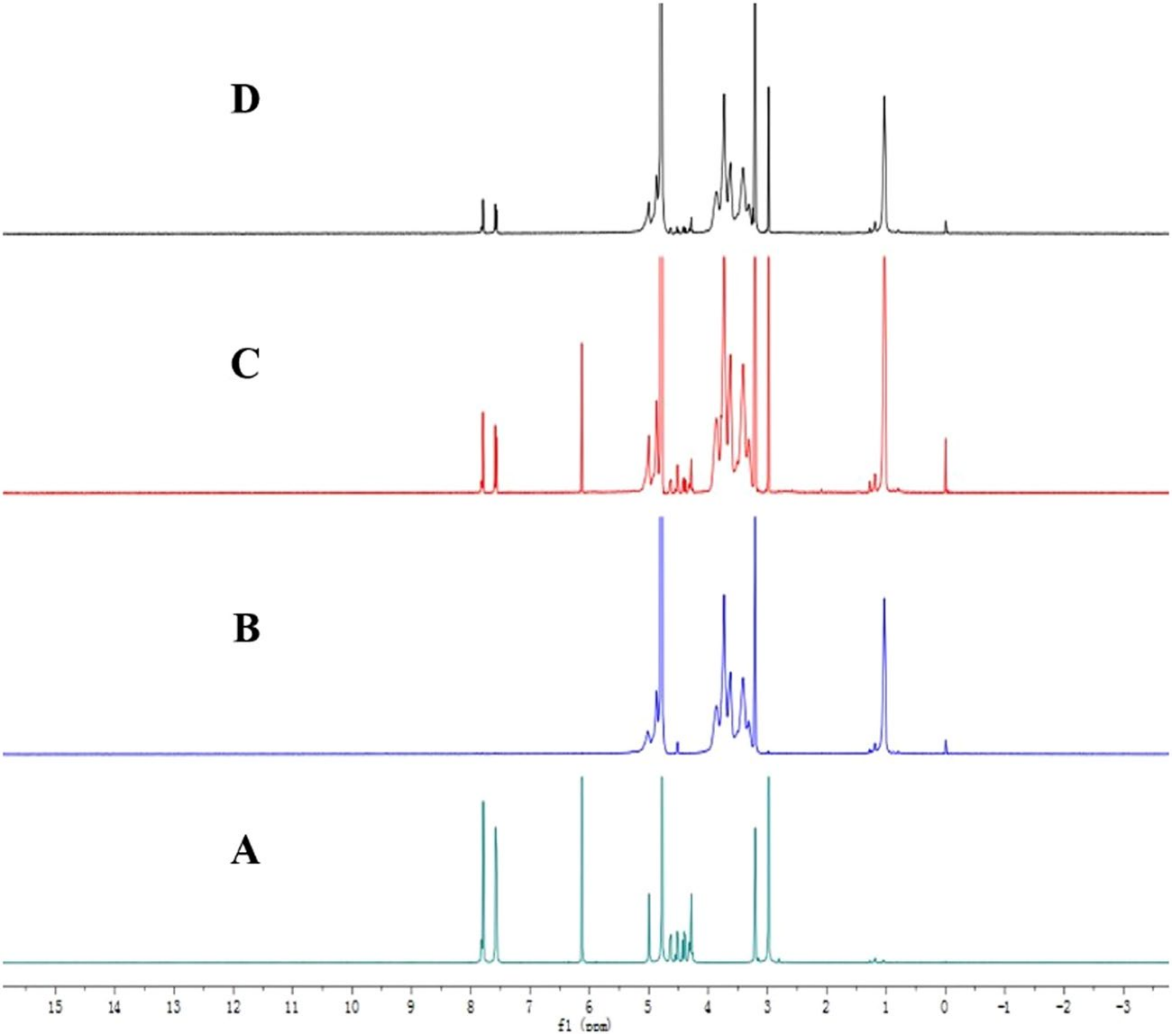
^1^H-NMR spectra of FF (**A**), HP-β-CD (**B**), PM (**C**), and FF-HP-β-CD (**D**).

**Figure 6:**
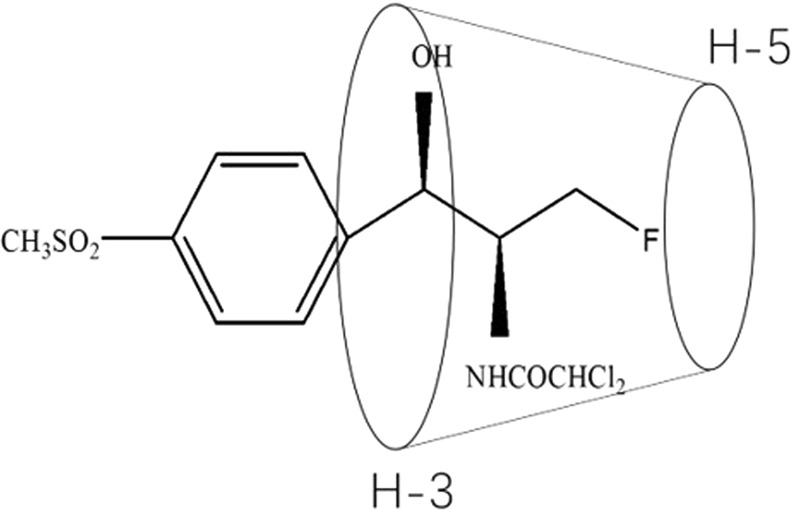
Proposed inclusion complex geometry of FF-HP-β-CD.

In this study, we performed docking studies of FF in the HP-β-CD cavity because the chemical and electronic environments of protons are affected during the complexation and are reflected through the changes in the *δ* values (Fig. 5). H-3 and H-5 are two atoms that form in the CD cavity. When FF enters the HP-β-CD, the hydrogen atoms in the FF and HP-β-CD cavity undergo corresponding changes due to the anisotropic shielding effect of the aromatic ring. From the comparison of the chemical shift values of HP-β-CD inclusion spectra with the FF-HP-β-CD, except for H-5, all the protons in the remaining CD positions apparently have a certain chemical shift, combined with the molecular structure of HP-β-CD. H-5 is at the narrow port, and H-3 is at the wide port. Thus, the FF molecules enter the HP-β-CD cavity from the wide mouth end. This result is consistent with the FTIR results. The comparison of the ^1^H-NMR spectra of FF and FF-HP-β-CD shows that the 8–11-position hydrogen atoms in the FF molecular structure all exhibit a 0.01–0.02 ppm chemical shift difference. Thus, FF is inferred to possibly be encapsulated in the HP-β-CD cavity from the side containing the dichloromethyl (–CHCl2) structure, whereas the FF sulfonyl side is outside the CD cavity (Fig. 6). These slight changes in chemical shifts indicate that the two are bound in a non-covalent bond, which is consistent with the principle of clathrate formation.

#### Muscle irritation test of FF-HP-β-CD injection

**Figure 7:**
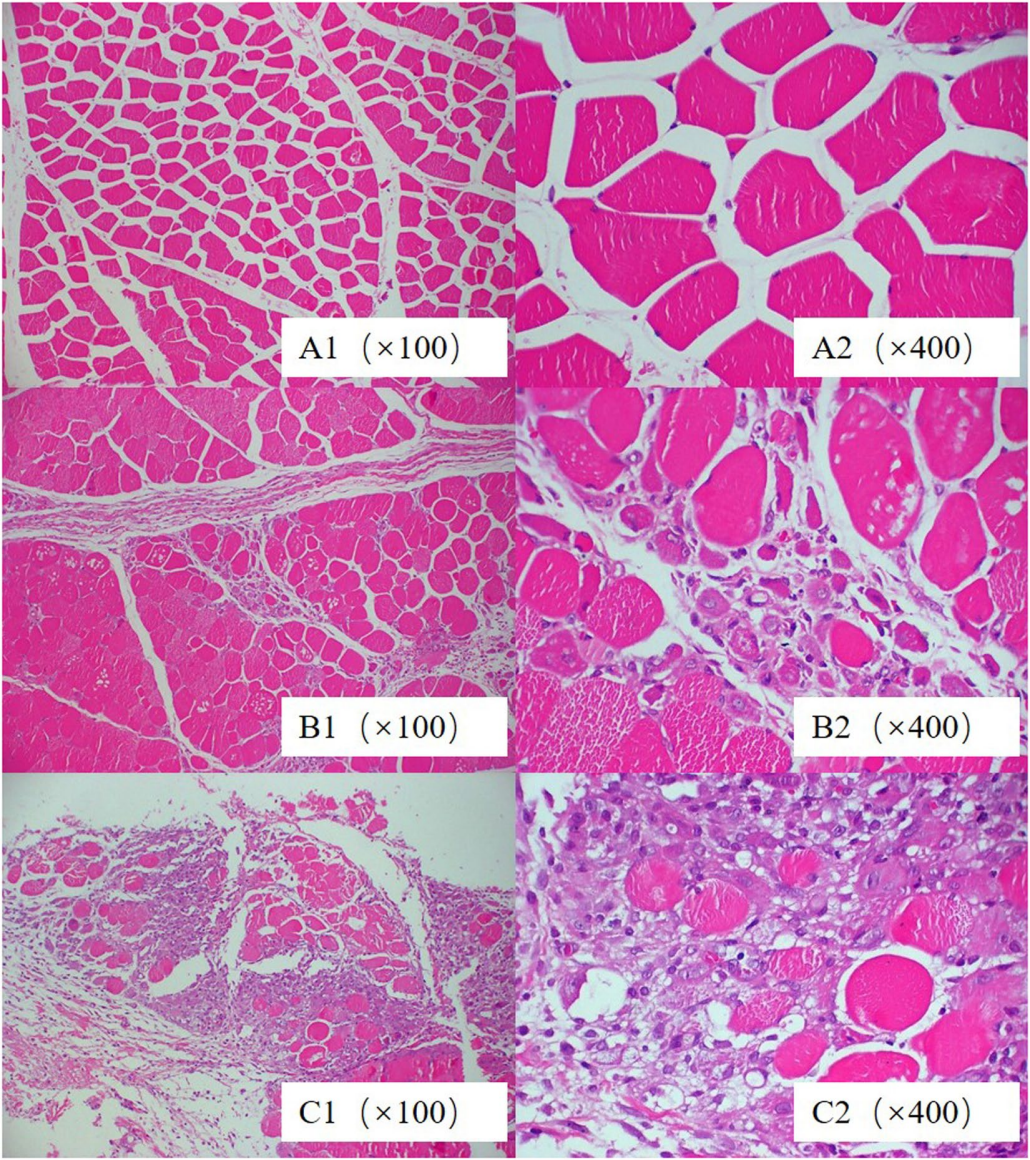
Pathological figures. Saline (**A1**,**A2**), FF-HP-β-CD (**B1**,**B2**), 10% FF injection (**C1**,**C2**).

A large demand exists for injections in the clinic, and the commonly used FF injection can cause local muscle irritation and toxicity. The current study commits to developing a water-soluble FF injection based on FF-HP-β-CD freeze-dried powder. Figure 7 illustrates the muscle histopathologic section. After continuous infusion for 3 days, saline has shown no visible sign of degeneration, necrosis, and inflammatory reaction; In addition, muscle interstitial cells has no hyperemia (Fig. 7A1,A2). One case (1/3) of FF-HP-β-CD aqueous solution group shows a small amount of muscle fiber atrophy and necrosis; no obvious fibrous tissue hyperplasia and inflammatory cell infiltration is observed (Fig. 7B1,B2). The clinically used 10% FF injection (Fig. 7C1,C2) presents a visibly large number of muscle fibers that are atrophied and necrotic and have a significantly large proliferation of fibroblasts. Histopathology shows that FF-HP-β-CD can significantly reduce the injection irritancy compared with 10% FF injection.

#### *In vivo* pharmacokinetic parameters in beagle dogs

**Figure 8:**
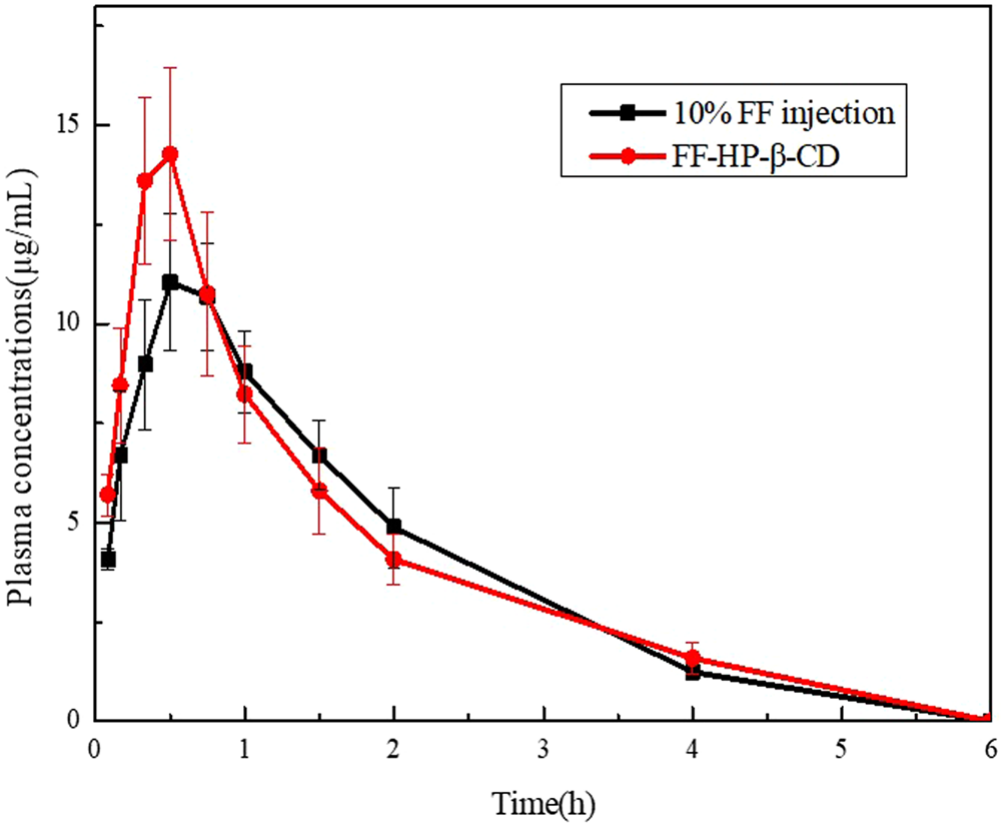
Plasma concentration–time curve of FF injection and FF-HP-β-CD solution.

The pharmacokinetic parameters of FF after intramuscular injection in beagle dogs were assessed by fitting a two-compartment open model to the individual concentration–time data for plasma. Figure 8 denotes the mean FF concentrations in plasma, and Table 1 presents the corresponding pharmacokinetic parameters. *t*_*1/2α*_, *Ka*, *V1/F*, and *T*_*max*_ are significantly lower than those of 10% FF injection (*P* < 0.05, *P* < 0.01). *t*_*1/2β*_, *K*_10_, *K*_12_, *K*_21_, and *C*_*max*_ are significantly higher than those of 10% FF injection (*P* < 0.05, *P* < 0.01).

**Table 1:** Pharmacokinetic parameters after the administration of 10% FF injection and FF-HP-β-CD solution (n = 6).

Pharmacokinetic parameters	10% FF injection	FF-HP-β-CD solution
t_1/2α_ (h)	0.77 ± 0.08	0.29 ± 0.07*
t_1/2β_ (h)	0.98 ± 0.19	1.92 ± 0.15*
Ka (1/h)	5.05 ± 0.25	3.66 ± 0.41*
V1/F (L/kg)	1.11 ± 0.21	0.72 ± 0.17**
CL/F (L/h/kg)	0.87 ± 0.14	0.81 ± 0.08
AUC_(0–6 h)_ (mg/L*h)	21.64 ± 2.38	22.13 ± 2.88
AUC_(0–∞)_ (mg/L*h)	23.65 ± 2.39	24.54 ± 2.84
K_10_ (1/h)	0.54 ± 0.023	1.16 ± 0.23**
K_12_ (1/h)	0.32 ± 0.10	0.96 ± 0.35**
K_21_ (1/h)	0.56 ± 0.02	1.04 ± 0.21*
C_max_ (mg/L)	11.56 ± 1.54	15.90 ± 3.15*
T_max_ (h)	0.58 ± 0.13	0.44 ± 0.09*

*P < 0.05, **P < 0.01, compared with 10% FF injection.

Because FF- HP-β-CD can increase the FF solubility, at the early stage, the dissolution of FF from HP-β-CD inclusion complexes is speeded up, resulting in the increase of drug absorption rate and peak plasma concentration. Therefore, after the FF-HP-β-CD injection was administered, the pharmacokinetic parameters, such as *C*_max_ and *T*_1/2α_, are all significantly different from those of 10% FF injection. The *C*_*max*_ of FF injection and FF-HP-β-CD in plasma of beagle dogs calculated in this study after 0.58 ± 0.13 h and 0.44 ± 0.09 h are 11.56 ± 1.54 mg/L and 15.90 ± 3.15 mg/L, respectively. *T*_1/2α_ are 0.77 ± 0.08 h and 0.29 ± 0.13 h for FF injection and FF-HP-β-CD. This indicates FF-HP-β-CD could avoid its common use with large dose. We can use lower dose of FF at the format of FF-HP-β-CD to reach a higher plasma concentration owing to its enhanced solubility. However, in the later stage, because FF is released from the cyclodextrin cavity, it will last longer in the body than the crude FF in the plasma^13^. That is why the elimination half-life (t_1/2β_) of FF-HP-β-CD is significantly longer than that of FF. Although the area under the concentration (AUC_0–∞_) of FF-HP-β-CD shows no significant increase in relation to FF, it has a larger area than that of FF. It indicates that FF-HP-β-CD increased bioavailability of FF to some extent.

The pharmacokinetics of FF has been previously studied in animal species, including rabbits, rats, dogs, carp, swine, and sheep^9,14–17^ Species differences in FF elimination exist. One-compartment open, two-compartment open, and non-compartment models exist after intravenous, intramuscular, and oral administrations in different species^18,19^. However, no pharmacokinetic report of FF or FF-HP-β-CD is available after intramuscular injection in beagle dogs. In our study, plasma concentration time data of FF-HP-β-CD and 10% FF injection after intramuscular injection in beagle dogs were adequately described by the two-compartment open model. The absorption of FF-HP-β-CD is faster and higher than FF, and its elimination of half-life is extended.

As we know, due to FF’s poor aqueous solubility, it is often used in large dose, which significantly limits its preparation and application. In addition, the commonly used FF injection is formulated with organic solvents. It can cause local muscle irritation and toxicity. Our study suggests FF-HP-β-CD freeze-dried powder, a water-soluble FF injection is a promising formulation for solving these problems and clinical application.

## Materials and Methods

### Materials

FF was obtained from Shanghai Yuanye Biotechnology Co., Ltd. (Shanghai, China); 10% FF injection was obtained from Guangdong Dahuanong Animal Health Products Co., Ltd. (Guangdong, China); HP-β-CD was obtained from Shandong Binzhou Zhiyuan Biotechnology Co., Ltd. (Shandong, China); and chromatographic-grade methanol and acetonitrile were purchased from Tianjin Kermel Chemical Reagent Co. Ltd (Tianjin, China). All the other chemicals and reagents were of analytical grade and used as received.

### Animals

All animal studies were approved by the Animal Ethical Experimentation Committee of Sichuan Agricultural University (SYXK[Chuan]2014-187), and the experimental procedures were in accordance with the national standard of the Laboratory Animal Requirements of Environment and Housing Facilities (GB 14925-2001) and the Sichuan Agricultural University Institutional Ethical Committee. Beagle dogs (10 ± 0.5 kg) were used for the pharmacokinetic study. New Zealand white rabbits (2.5 ± 0.2 kg) were used for the muscle irritation tests. All the animals were supplied by the Experimental Animal Center of Sichuan Agricultural University (Sichuan, China). Before the experiment, the animals were acclimatized at 25 °C ± 2 °C under natural light/dark conditions for 1 week with free access to food and water.

### Preparation of FF-HP-β-CD

FF-HP-β-CD was prepared using the solution-stirring method. The single-factor experiment and orthogonal design were adopted in the optimization of the prescription and manufacture process of FF-HP-β-CD. In brief, FF and HP-β-CD (mole ratio of 2:1) were weighed, and FF was dissolved with 50% methanol. HP-β-CD was prepared in a 20% (w:v) aqueous solution. Then, the FF organic solution was added to the HP-β-CD aqueous solution. The pH of the mixture was adjusted to 5 and stirred at 100 r/min at 30 °C for 2 h. The inclusion complex solution was transferred to a rotary evaporator (Shanghai Yarong Biochemistry Instrument Factory, Shanghai, China), and the organic solvent was evaporated at 65 °C. Subsequently, the FF-HP-β-CD solution was obtained. Finally, the FF-HP-β-CD solution was filtered with a 0.22 μm microporous membrane and was lyophilized. The lyophilization process was as follows: pre-frozen at −30 °C for 4 h and then freeze dried at −50 °C for 16 h.

### Characterization of FF-HP-β-CD

The characterization of FF-HP-β-CD was studied via SEM (SU8020, Hitachi, Japan), DSC (METTLER TOLEDO, Switzerland), XRD (Bruker D8 Advance, Germany), FTIR (Spectrum Two, PerkinElmer, USA), and ^1^H-NMR (Bruker AV, Bruker, Germany).

### Muscle irritation test of FF-HP-β-CD injection

Six rabbits were randomly divided into two groups, and the control group rabbits were administered with 20 mg/kg of 10% FF injection. The test group rabbits were injected with 20 mg/kg of FF-HP-β-CD aqueous solution (lyophilized powder was dissolved in a sterile saline) in the left leg muscle. Intramuscular injection of equal volume of sterile saline was administered on the right side. Continuous injection was administered for 3 days. Rabbits were sacrificed 48 h after the last dose; then, the muscle at the injection site was isolated and fixed in 10% neutral carbonated-buffered formaldehyde. The fixed tissues were paraffin-embedded and sliced using a microtome. Hematoxylin and eosin-stained sections of the muscle tissues were evaluated.

### *In vivo* pharmacokinetic studies

A pharmacokinetic study was performed on healthy beagle dogs. Twelve beagle dogs were randomly divided into two groups. The control group was administered with 10% FF injection, whereas the other was administered with FF-HP-β-CD aqueous solution at the left hind limb muscle (20 mg FF/kg body weight)^9,14^. At predetermined time points, blood samples were collected from the auricular vein. Samples were centrifuged at 3000 rpm for 10 min within 1 h after sampling, and plasma was collected and stored at −70 °C until analysis. FF was extracted from the plasma through liquid–liquid extraction with acetonitrile. In brief, the samples were prepared by adding 0.4 mL of plasma to 1.2 mL of acetonitrile. The mixture was vortexed for 3 min and centrifuged at 12000 rpm for 10 min. After filtering through the cellulose acetate membranes of 0.22 μm pore diameters, 20 μL of the collected filtrate was injected into the high-performance liquid chromatography (HPLC) for analysis.

All samples were analyzed on a Shimadzu LC-2010CHT system (Shimadzu Corporation, Kyoto, Japan) with a UV detector. HPLC analysis was performed using a 5 μm C18-silica-based stainless steel Kromasil column (4.6 mm × 250 mm, Akzo Nobel, Bohus, Sweden) as a stationary phase. The mobile phases were acetonitrile: 0.1% of the phosphoric acid solution (35:65, v:v) with a 0.5 mL/min flow rate. Then, the drug concentration–time data in plasma were fitted using DAS 2.0 software supplied by the Pharmacological Society of China (Beijing, China). The most appropriate pharmacokinetic model was evaluated in terms of the range of the coefficient of determination (*r2*) and comparisons of Akaike’s information criterion values.

### Statistical analysis

The statistical analysis of data was performed using analysis of variance by using SPSS 15.0 software (SPSS Inc., Chicago, USA). The results were expressed as a mean ± standard deviation. A difference of p < 0.05 was considered statistically significant.
